# Cognitive Assessment Test: Validation of a Short Cognitive Test for the Detection of Mild Cognitive Disorder

**DOI:** 10.1155/2018/3280621

**Published:** 2018-07-02

**Authors:** Kelly Estrada-Orozco, Kely Bonilla-Vargas, Francy Cruz, Oscar Mancera, Miguel Ruiz, Laura Alvarez, Rodrigo Pardo, Humberto Arboleda

**Affiliations:** ^1^Clinical Research Institute, National University of Colombia, Colombia; ^2^Neurosciences Group, National University of Colombia, Colombia; ^3^Institute of Genetics, National University of Colombia, Colombia

## Abstract

**Introduction:**

Cognitive disorders are a clinical and research challenge; in particular, the mild cognitive disorder (MiCD) requires diagnostic suspicion and tools with adequate performance for its detection. The objective of this study was the validation of a short cognitive test (CATest) for the detection of MiCD in population of 50 years or more.

**Methods:**

A diagnostic accuracy study was assembled and performed in a prospective cohort. A consecutive sample of 200 Colombian subjects who represented the whole spectrum of the condition of interest allowed us to reach the objective. Validity was determined by concurrent criteria. The cut points were determined by the ROC curves considering the best overall performance and accuracy of the test.

**Results:**

CATest was validated to detection of MiCD at a cut-off point of 18. As a result, scores lower than 18 classified the participants as MiCD. At this cut-off point, CATest showed sensitivity of 84.3% (CI 76 to 90.16), specificity of 71.4% (**CI** 95% 61.8 to 79.43), positive predictive value of 75% ( 95% CI 66.79 to 82.42), and area under curve AUC 0.8518 (standard error SE 0.0265).

**Discussion:**

CATest has an adequate performance as a short cognitive test for the detection of MiCD. Its performance is superior to MiniMental and similar to Montreal Cognitive test (MoCA) according to the data reported in the literature. The advantages over other tests are the evaluation of all cognitive domains, time of application, and easy interpretation of results. CATest is a free use alternative for MiCD detection.

## 1. Introduction

Aging of population is one of the issues that most concerns the health system [1], due to the numerous comorbidities that accompany this population [2], the consumption of resources derived from their care, and the high burden of disease in terms of disability from many of these diseases.

Dementia represents a high burden of disease that mainly affects the over 65s [1, 3]. In recent years, dementia as a term has been replaced by major cognitive disorder due to the high stigma associated with this disease.

During the aging process, changes occur within the cognitive domains, many of which go unnoticed, especially if the changes are small and demands on the environment in which the patient operates are small. Other changes, on the contrary, obligate the individual to generate a series of strategies aimed at compensating for the faults observed in the activities of his or her daily life.

Cognitive disorders are classified according to the Diagnostic and Statistical Manual of Mental Disorders DSM 5 [4] in mild neurocognitive disorder (MiCD), if the alteration in the higher brain function is not so pronounced as to generate difficulties in the activities of the individual, and major neurocognitive disorder (MCD) (a term that replaces dementia included until the previous version of the DSM IV), if the alteration of these functions affects their functionality at work, social, and/or family level. The definition includes a loss of these higher brain functions, after they have had a habitual development throughout life.

According to the latest pronouncement of the World Alzheimer Report 2015 [3] which details the global prevalence of dementias, a total of 46.8 million people living with MCD were estimated for the middle of 2015. On the other hand, MiCD affects between 3% and 20% of adults over 65 [5–7]; in other studies, prevalence greater than 22% has been found [8–13] and the prognosis in general practice is variable: approximately 25% of people develop MCD within three years after diagnosis, but about 40% return to normal [14].

Evidence on factors that are related to the progression from mild cognitive disorder to major cognitive disorder is becoming more common [15–17], and many of these factors that have been identified are largely modifiable [18]. This new evidence, among many other reasons, allows the consideration of mild cognitive disorder to be of vital importance at a clinical and social level.

Diagnosis of cognitive disorders is important especially in the early stages because many of its causes are potentially reversible such as depression, side effects of medication, excess alcohol, thyroid disease, vitamin deficiencies, and sleep disorders. In addition, even in the case of primary neurodegenerative disorders, early detection allows mitigate factors that are known to lead to more rapid progression of the disease [15, 16], and this mitigation will ultimately slow progression.

Another benefit of early disease detection that is often overlooked is the extra time an individual will have to arrange their financial and legal obligations regarding end of life care [19].

Science also does not overlook the benefits of early and accurate screening, as it also allows affected people to decide whether or not to participate in clinical trials, including experimental therapy trials that can slow or stop the progression of the disease [19, 20], a field that has worked for many years with no conclusive results so far.

At present there are a large number of studies available that represent adequate evidence on the diagnosis of major cognitive disorder. The challenge for health professionals and science lies in the beginning phases of this disorder that go unnoticed for the subject and health care personnel. This limits the ability to predict the emergence of a syndrome of greater complexity and the potential of a high degree of disability for the individual in the future.

There are tools that have been validated to detect neurocognitive disorder [21–27]; however the target population is over 65 years and accuracy and reliability present enormous variability to diagnostic MiCD. The above can be explained by the differences in population where it has been applied, diagnostic criteria to define the condition, and complexity and scope of the tools.

The aim of this study was validate a tool (cognitive assessment test (CATest)). for the detection of mild cognitive disorder in subjects aged 50 years or older, taking from the literature diagnostic elements with the highest level of diagnostic accuracy for this population, which contribute to a greater operative performance of the tool.

## 2. Methods

### 2.1. Design of Study

Diagnostic test accuracy study assembled in a prospective cohort.

### 2.2. Participants

A cohort with a total of 200 consecutive Colombian participants enrolled in the National University of Colombia Clinic of Dementia was included. Inclusion criteria were (1) age equal to or greater than 50 years, (2) at least 1-year education, and (3) adequate vision and hearing to complete neuropsychological (NP) testing. Exclusion criteria were (1) history of severe brain trauma; (2) lifetime history of schizophrenia, manic-depressive disorder, or schizoaffective disorder; (3) current alcohol or drug abuse/dependence; (4) obstructive sleep apnea syndrome; and (5) significant disease or unstable medical condition (i.e., chronic renal failure, chronic hepatic disease, or severe pulmonary disease) and thyroid disease with no hormonal substitution.

### 2.3. Sample Size

Sample size was calculated in 200 patients; the parameters used in the calculation were prevalence of cognitive disorder 40%, sensitivity 90% or higher, and specificity 80% or higher.

### 2.4. Medical Evaluation

A neurological clinical assessment was performed. The review of personal clinical history, mental and neurological examination, cognitive screening tests (MiniMental MMSE 2 [28], Neuropsychiatric Inventory [29]), and functionality scales (Lawton and Brody Scale [30, 31]) was completed, as well as review of tests such as lipid profile, glucose, thyroid tests, levels of vitamin B12 and folic acid, tests of hepatic and renal function, and serology VDRL.

In the participants with abnormal results in cognitive screening tests, a brain image was requested by magnetic resonance and reviewed in a consultation during follow-up.

### 2.5. Neuropsychological (NP) Evaluation

Neuronorm-Col [32] diagnostic NP battery consisted of tests of (1) language tests (Boston Naming Test, Token Test), (2) visuoconstructive skills (Rey-Osterrieth Complex Figure), (3) attention and executive functions (WAIS-III Digit Retention tests, Corsi Cubes, trail making test A and B (TMT A and B), digit-symbol test (SDMT), Stroop color word Test, Tower of London test, Win- dingo Card Sorting Test and Verbal Fluency), and (4) memory (Free and Cued Selective Reminding Test).

### 2.6. Diagnostic Classification of the Participants

Cognitive classification was determined via a multidisciplinary consensus meeting including (neurologist, neuropsychologist, and neuroscientist); criteria to classification of cognitive disorder from DSM 5 [4] were used and NP testing, medical and social history, daily functioning, reported cognitive symptoms, and neuroimaging findings were reviewed.

#### 2.6.1. Normal Performance

Criteria for normal performance were (1) no more than one test score lower than expected within a cognitive domain and (2) no more than two scores lower than expected across domains, with the threshold corresponding to 1.0 standard deviation (SD) below age adjusted control means.

#### 2.6.2. Cognitive Disorder (CD)

NP criteria for MiCD included scores on at least two individual tests within a cognitive domain, greater than 1.0 SD below education and age-corrected. MCD included scores on at least two individual tests within a cognitive domain lower than 2.0 SD.

### 2.7. Cognitive Assessment Test (CATest)

The construction of CATest is the result of a systematic review performed by the National University of Colombia group of neurosciences.

CATest includes the following.

The* immediate recovery test*: it consists of a list of 5 words, which allows evaluating episodic short-term memory and attentional functions during the first trial. In the test, the subject is asked to repeat 5 words during two trials, and after a short period of time, with distracting elements, he is asked to remember the 5 words. The recovery must be done spontaneously.

The* clock drawing test*: it evaluates different cognitive skills, including attention, visuospatial abilities, abstract conceptualization, and executive control. During the drawing test of the clock, the participants are asked to draw a clock that has all its parts (circumference, hour hand, minute hand and second hand, and numbers) and indicate on it the time 11:10. The drawing of the circumference, the numbers in correct position and order, and the location of the requested time are qualified. There are no time limits to complete it.

The* phonological fluency test*: it is applied in a time of 1 minute; it has a restrictive character of phonological type, for the production of words limiting the beginning of the same to a letter that is indicated when giving the instruction of the test.

CATest utilizes 2 letters: “M” or “P”, and the double selection is done to prevent learning bias during the serial application.

CATest is rating from 0 to 21, considering 15 points to memory evaluation (Supplementary materials (available here)).

### 2.8. Analysis

Baseline distributions of the demographics, marital status, and education were presented according to the distribution of normality of each variable. A subgroup analysis by diagnostic (normal performance, MiCD, and MCD) was presented.

The receiver operational characteristic (ROC) curve analysis [33] was utilized to characterize the performance of the CATest in distinguishing MiCD patients from normal healthy controls and MiCD patients from MCD patients.

Optimal cut-off point was determined from a sensitivity analysis of the operative characteristics of the test; it included sensitivity, specificity, positive predictive value, negative predictive value, true positive (TP), and false positive (FP) rates, Likelihood ratio (LR + and LR -) as well as diagnostic odds ratio (DOR).

Operative characteristics of each cut-off evaluated in the sensitivity analysis were modulated considering the proportion of accurately classified patients and the cost of making a false positive mistake or a false negative mistake. As criteria to define it, FP rate was maximized for MiCD and TP was maximized for MCD.

The results were presented with the 95% confidence interval.

Data were analyzed with statistical software STATA ® V.13.

## 3. Results

A total of 339 participants were evaluated between March 2016 and November 2017. 109 were excluded (22 active psychiatric disease, 87 other causes: hearing loss, Parkinson disease, history of severe brain trauma, and cognitive disorder since childhood), and 30 participants did not complete the neuropsychological test. 200 participants were included.

The prevalence of cognitive disorders in the sample was 51% (95% CI 44.1-57.9) with a prevalence of 32% (CI 95% 25.5-38.5) for MiCD and 19% (CI95% 13.6-24.4) for MCD.

The study sample consisted predominantly of women (67%), average age of the participants were between 53 and 66 years (SD 8.84), and there were no differences between the ages by sex in the study with men being on average 66.65 years (95% CI 64.25-69) and women 66.5 years (95% CI 65-67.9) (P 0.9105).

The educational level measured as the median of years of schooling was 16, Rank (1-29), and 45% of the participants were married (Table 1).

According to diagnostic category, statistical differences were not found in age of participants in normal performance group and MiCD ( average 64.8 and 65.5 year, respectively); however, the age in MCD group presented statistical differences ( 72.6 (CI95% 69.2-75.9)) (Table 2).

Women represented the highest proportion in the groups of normal performance subjects and MiCD, but this trend was not observed in the group of patients with MCD (women 34.21%) (P < 0.0001).

Years of schooling also proved to be a variable that differentiated the groups (median of 11 years in MCD group and 16 years in normal performance and MiCD groups) (P 0.0001).

**Figure 1:**
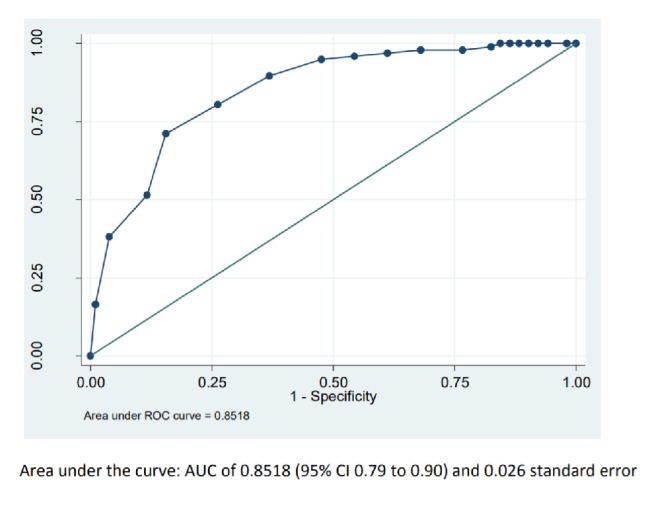
ROC curve and value of correct classification (AUC).

ROC curves were developed (Figure 1) to select the most accuracy cut-off point (normal performance, MiCD). After the sensitivity analysis, CATest was validated for the detection of MiCD at a cut-off point of 18. As a result, scores lower than 18 classified the participants as MiCD. At this cut-off point, CATest showed sensitivity of 84.3% (CI 76 to 90.16), specificity of 71.4%, (CI 95% 61.8 to 79.43), positive predictive value of 75% (95% CI 66.79 to 82.42), and area under curve AUC 0.8518 (standard error SE 0.0265) (Table 3).

As CATest was validated in a sample of patients with cognitive disorder (MCD, MiCD, and normal performance); a second ROC curve was developed (Figure 1) to select the cut-off point to classify the participants as MiCD from the cognitive disorder sample. The most accuracy cut-off for this goal was 14. The CATest accuracy at this point was 88.5 95% CI (83.33-92.21) and AUC 0.95 (Table 3).

CATest time application was calculated in the study sample, an average of 3 minutes and 55 seconds (SD 54 seconds). Time of application was varied from analysis by diagnostic groups (normal performance 3 minutes, MiCD 4 minutes and 42 seconds; MCD 6 minutes, SD 59 seconds).

## 4. Discussion

As a result of this study we obtain the validation of a new short cognitive test for the detection of MiCD in population with 50 years and older. For screening context, CATest has sensitivity of 84.3% (95% CI 76 to 90.16), specificity of 71.4% (95% CI 61.80 to 79.43), and accuracy of 0.84, which classifies it as a test of moderate accuracy.

CATest accuracy can be better (accuracy 0.95) if the purpose is to classify a patient from a population with cognitive disorder (MCD and MiCD); that is, when the objective of the test is to classify the degree of cognitive disorder.

In relation to the characteristics that are attributed to a short test [34], characteristics of sensitivity and specificity above 80% are desired, which according to the confidence intervals of CATest is met in this study. Another important characteristic is the accuracy that the previous study suggests should be greater than 0.8 and it is also true for the validated test that the value of the desired accuracy is enclosed within the 95% confidence limits calculated for the general population from the sample. Although the performance of the test is not the performance of a perfect test, we found that CATest has better performance for detection of MiCD than MiniMental (pooled sensitivity less than 70%, accuracy 0.73) [35–37], which is the most recognized cognitive test [22] and others reported in the literature(test your memory, ACE/ACE-R, CAMCOG) [37] and similar performance to MoCA test [25, 36–38].

A recent meta-analysis [37] aimed at finding and measuring the diagnostic accuracy of short cognitive tests published in the literature found 9 different cognitive tests, among them MMSE, MoCA, clock drawing test, and recall test had the major number of studies and participants; the meta-analysis qualified as having good methodological quality according to the AMSTAR 2 tool reports that the recall tests have the best overall accuracy given by a sensitivity(S) 89% and a specificity (Sp) of 84%. Despite the performance of this test, it has the difficulty of evaluating only the memory domain. Amnesic cognitive disorders correspond to only 60% of cognitive disorders, so the use of this unique test would be less sensitive if the detection of other types of cognitive disorders is sought.

In relation to MoCA, CATest presents a similar performance(S: 83% and Sp: 75%) [37]; however, CATest has an advantage on MoCA as a result of the application time (4 minutes for CATest versus 10 minutes for MoCA [39]), which would facilitate the use of CATest in primary care settings.

The main strength of this study is the homogeneity of the diagnostic criteria that were defined a priori, as well as the fact that the study was assembled in a cohort, with strict selection criteria for its participants, which reduces the probability of including biases, ensuring internal validity.

In contrast, the special susceptibility of diagnostic test studies to the location where they are developed is known by reports in the literature, since the characteristics of the study sample are usually different from those of the general population, compromising external validity. In our study, a Colombian reference center in attention of cognitive disorder was the location; however, preventive measures of this selection bias were controlled with rigorous design, achieving that the sample will be made up of volunteers from a public call in the media. One of the main limitations of this study is the high prevalence of CD in the sample, since it has been reported that it can behave as a bias that directs the results of accuracy towards over estimation [40]. Therefore, the results of this study should be interpreted with caution in scenarios with lower prevalence, as well as with populations with lower educational levels than those of our sample, since the measurement of cognitive performance is especially vulnerable to this type of characteristics.

Another aspect that should be considered in the interpretation of the results of sensitivity and specificity is the value corresponding to the margin of error that is evident in the wide of the confidence intervals and that are a consequence of the size of the sample used for the validation of the CATest.

Future studies are necessary to verify the performance results of CATest in different scenarios (educational level, etiology of cognitive disorder, CD prevalence, and populations from other nationalities) to determine its reliability, as well as studies of direct comparisons with other tests of a similar nature, in order to obtain more precise results.

In conclusion, CATest is an alternative with adequate performance for the detection of cognitive disorder after 50 years. The ease of qualification and the short application time make it an attractive proposal for primary care scenarios. Additionally, the free use of restrictions makes CATest a useful alternative in daily clinical practice, educational, and research scenarios.

## Figures and Tables

**Table 1:** Characteristics of the participants.

	**TOTAL**		**Men**		**Women**		
	**N=200**	**CI 95**%	**n= 66**	**CI 95**%	**n= 134**	**CI 95**%	**P Value**
**Age (Years)** ^*∗*^	66.53( 8.84)	(65.3-67-7)	66.65 (9.94)	(64.25-69)	66.5(8.28)	(65-67.9)	0.9105
**Education (Years)** ^*∗∗*^	16(1-29)		16(1-29)		15(2-25)		0.5183

	**n/200 (**%**)**	**CI 95**%	**n/66 (**%**)**	**CI 95**%	**n/134**	**CI 95**%	

**Civil Status**							
** Married**	90(45)	(38.1-51.9)%	39(59)	(47.2-71)%	51(38)	(29.8-46.3)%	0.0050
** single**	28(14)	(9.2-18.8)%	7(10.6)	(6.5-23.8)%	21(15.67)	(9.5-21.8)%	0.3312
** widower**	24(12)	(7.5-16.5)%	2(3)	(0-7.2)%	22(16.41)	(10.1-22.7)%	0.0060
** Divorced**	17(8.5)	(4.6-12.4)%	2(3)	(0-7.2)%	15(11.19)	(5.9-16.5)%	0.0507
** No information**	41(20.5)		*∗*-*∗*		*∗*-*∗*		

^*∗*^Mean (standard deviation (SD)). ^*∗∗*^Median (range).

**Table 2:** Characteristics of the participants by diagnosis category.

	**TOTAL**		**Normal performance**		**MiCD**		**MCD**		
	**N=200**	**IC 95**%	**n= 98**	**IC 95**%	**n = 64**	**IC 95**%	**n = 38**	**IC 95**%	**P value**
**Age (years)** ^*∗*^	66.54(8.86)	(65.3-67-7)	64.83( 7.487)	(63.34-66.31)	65.53(8.32)	(63.49-67.56)	72.6(10.48)	(69.22-75.96)	**< 0.001**
**Women**	134(67%)	(60.5-73.5)	75(76%)	(68.1-84.5)	45(70.31%)	(59.1-89.5)	13(34.21%)	(19.1-49.3)	**< 0.001**

** Education (years)** ^*∗∗*^	16(1-29)		16(4-24)		16(3-25)		11(1-29)		**0.001**

**Category **	**(**%**) n/200**	**CI 95**%	**(**%**) n/98**	**CI 95**%	**(**%**) n/64**	**CI 95**%	**(**%**) n/38**	**CI 95**%	**< 0.001**

**< 5 years**	22(11)	(6.7-15.3)	4(4)	(0.2-8)	2(3.1)	(0-7.4)	16(42.1)	(26.4-57.8)	
**>5 - 11 years**	35(17.5)	(12.2-22.8)	19(19.38)	(11.6-27.2)	13(20.31)	(10.5-30.2)	3(7.9)	(0-16.5)	
**>11-16 years**	69(34.5)	(27.7-41.1)	32(32.65)	(23.4-41.9)	24(37.5)	(25.6-49.4)	13(19.1)	(19.1-49.3)	
**>16 years**	74(37.5)	(30.3-43.7)	43(43.87)	(34.1-53.7)	25(39)	(27.1-51)	6(15.78)	(4.2-27.4)	

MiCD: mild cognitive disorder and MCD: major cognitive disorder.^*∗*^Mean (standard deviation (SD)). ^*∗∗*^Median (range).

**Table 3:** CATest performance at cut-off point.

**Cut-off point-* *->**	**14**		**18**	
		**CI 95**%		**CI 95**%
**Sensitivity**	86,80%	72,67 - 94,24	84,30%	76 - 90,16
**Specificity**	88,90%	83,12 - 92,85	71,40%	61,80 - 79,43
**Positive predictive value**	64,70%	50,98 - 76,36	75,40%	66,79 - 82,42
**Negative predictive value**	96,60%	92,38 - 98,55	81,40%	71,89 - 88,21
**Proportion of false positives**	11,10%	7,14 - 16,8	28,60%	20,56 - 38,19
**Proportion of false negatives**	13,20%	5,75 - 27,32	15,70%	9,89 - 23,97
**Accuracy**	88,50%	83,33 - 92,21	78,00%	71,76 - 83,18
**Diagnostic odds ratio**	52,8	18,28 - 152,48	13,44	6,73 - 26,8
**Youden's J index**	0,8	*∗∗*	0,6	*∗∗*
**Likelihood ratio LR (+)**	7,82	4,96 - 12,29	2,95	2,13 - 4,08
**Likelihood ratio LR (-)**	0,15	0,06 - 0,33	0,22	0,13 - 0,34

## Data Availability

The data used to support the findings of this study are included within the article.

## Supplementary Materials

Supplementary Materials Supplementary file 1: cognitive assessment test (CATest) instrument English version. Supplementary file 2: cognitive assessment test (CATest) instrument Spanish version.
