# Supplementary material for: Cognitive Assessment Test: Validation of a Short Cognitive Test for the Detection of Mild Cognitive Disorder
Source: Int J Alzheimers Dis. 2018 Jul 2;2018:3280621. doi: 10.1155/2018/3280621 (PMC6051079; doi:10.1155/2018/3280621)
Supplement: Supplementary Materials — Supplementary file 1: cognitive assessment test (CATest) instrument English version. Supplementary file 2: cognitive assessment test (CATest) instrument Spanish version. [file 3280621.f1.docx]

Supplementary material

| CaTest English version | |  |  |  |  |  |  |  |
| --- | --- | --- | --- | --- | --- | --- | --- | --- |
| **Cognitive Assessment Test - CATEST** | | | | | | | | |
|  |  |  |  |  |  |  |  |  |
| **Name______________________ID_______** | | | |  | **Age:** |  |  |  |
|  |  | **Date:** |  |  |  |  |  |  |
| **1.       Registration of information** | | |  |  |  |  |  |  |
| **The 5 words are read in order then the patient is asked to remember the words. If all the words are not remembered, the words are read a second time. When finished, the patient is asked to say the words on the list.** | | | | | | | | |
|  | **FACE** | **SILK** | **CHURCH** | **CARNATION** | **RED** | **TOTAL** | |  |
| **REPETITION 1** |  |  |  |  |  | **___/5** | |  |
| **REPETITION 2** |  |  |  |  |  | **___/5** | |  |
| **2. Draw a clock that is marking 11:10** | | | | | | **___/3** | |  |
|  |  |  |  |  |  |  |  |  |
|  |  |  |  |  |  |  |  |  |
| **3. Verbal fluency** | |  |  |  |  |  |  |  |
| **Aplication 1: Letter P** | |  | **Aplication 2: Letter M** | |  | **___/3** | |  |
|  |  |  |  |  |  |  |  |  |
|  |  |  |  |  |  |  |  |  |
|  |  |  |  |  |  |  |  |  |
|  |  |  |  |  |  |  |  |  |
| **1.b Recovery** | **FACE** | **SILK** | **CHURCH** | **CARNATION** | **RED** | **TOTAL** | |  |
|  |  |  |  |  |  | **___/5** | |  |
|  |  |  |  |  | TOTAL | **___/21** | | |
|  |  |  |  |  |  |  |  |  |

| CaTest Espanish version | |  |  |  |  |  |  |  |
| --- | --- | --- | --- | --- | --- | --- | --- | --- |
| **Cognitive Assessment Test - CATEST** | | | | | | | | |
|  |  |  |  |  |  |  |  |  |
| **Nombre______________________Documento_______** | | | |  |  |  |  |  |
| **Edad:** |  | **Fecha:** |  |  |  |  |  |  |
| **1.       Registro de la información** | | |  |  |  |  |  |  |
| **Se leen en orden las 5 palabras a continuación, al finalizar se le pide al paciente que recuerde las palabras. En caso de no recordarlas todas, se leen una segunda vez, al terminar se pide al paciente que diga las palabras de la lista.** | | | | | | | | |
|  | **ROSTRO** | **SEDA** | **IGLESIA** | **CLAVEL** | **ROJO** | **TOTAL** |  |  |
| **REPETICION 1** |  |  |  |  |  | **___/5** |  |  |
| **REPETICION 2** |  |  |  |  |  | **___/5** |  |  |
| **2. Dibujar un reloj que marque las 11:10** | | | | | | **___/3** |  |  |
|  |  |  |  |  |  |  |  |  |
|  |  |  |  |  |  |  |  |  |
| **3. Fluidez verbal** | |  |  |  |  |  |  |  |
| **Aplicación 1: Letra P** | |  | **Aplicación 2: Letra M** | |  | **___/3** |  |  |
|  |  |  |  |  |  |  |  |  |
|  |  |  |  |  |  |  |  |  |
|  |  |  |  |  |  |  |  |  |
|  |  |  |  |  |  |  |  |  |
| **1.b Recobro** | **ROSTRO** | **SEDA** | **IGLESIA** | **CLAVEL** | **ROJO** | **TOTAL** |  |  |
|  |  |  |  |  |  | **___/5** |  |  |
|  |  |  |  |  | TOTAL | **___/21** | | |
|  |  |  |  |  |  |  |  |  |
